# Tumor circulating DNA profiling in xenografted mice exposed to intermittent hypoxia

**DOI:** 10.18632/oncotarget.2785

**Published:** 2014-11-16

**Authors:** Rene Cortese, Isaac Almendros, Yang Wang, David Gozal

**Affiliations:** ^1^ Department of Pediatrics, Section of Pediatric Sleep Medicine, Pritzker School of Medicine, The University of Chicago, Chicago, IL

**Keywords:** Obstructive sleep apnea, Intermittent hypoxia, Circulating DNA, Xenograft, Epigenetics, DNA methylation

## Abstract

Intermittent hypoxia (IH) a hallmark characteristic of obstructive sleep apnea (OSA), is proposed as a major determinant of processes involving tumor growth, invasion and metastasis. To examine whether circulating DNA (cirDNA) in blood plasma reflects changes in tumor cells under IH conditions, we used a xenografted murine model. Mice engrafted with TC1 epithelial lung cancer cells and controls were exposed to IH or room air (RA) conditions. Plasma cirDNA amounts were significantly increased in mice exposed to IH (p<0.05). Significant associations between plasma cirDNA concentrations and tumor size, weight and invasiveness also emerged (p<0.05). Using a methylation microarray-based approach, we identified 2,094 regions showing significant differential cirDNA modifications. Systems biology analyses revealed an association with molecular pathways deregulated in cancer progression and with distal and TSS-associated transcription factor binding sites. We detected clusters of highly variable regions in chromosomes 7, 13, 14 and X, which may highlight hotspots for DNA deletions. Single locus displayed high intragroup variation, suggesting cellular heterogeneity within the tissue may be associated to cirDNA release. Thus, exposures to IH increase the shedding of cirDNA into circulation, which carries epigenetic modifications that may characterize cell populations within the tumor that preferentially release their DNA upon IH exposure.

## INTRODUCTION

Sleep disorders in general, and more particularly obstructive sleep apnea (OSA), have been associated with accelerated cancer progression, aggressiveness, and mortality [1, 2]. OSA is a highly prevalent disorder that occurs in all age groups and both sexes with an estimated prevalence of 4 % to 10 % in adults [3]. This disorder is characterized by repetitive obstructions of the upper airway during sleep that result in intermittent hypoxia (IH), increased inspiratory efforts, repetitive arousals from sleep to reestablish respiration leading to sleep fragmentation (SF) and episodic elevations in the concentrations of blood CO_2_ [4, 5]. In the last 2 decades, it has become apparent that numerous and serious end-organ morbidities are associated with OSA and virtually affect all organ systems, including cancer [1-10]. The mechanisms potentially leading to the cardiovascular [11-14], cognitive [15-17], and metabolic [18-20] morbidities of OSA have been extensively studied in human and animal models, with IH being proposed as a major determinant of the processes involving tumor invasion and metastasis [21, 22]. Indeed, we have recently shown that tumor growth, cell proliferation, migration and invasiveness are all increased in murine models of OSA, being selectively exposed to either IH [23] or SF [24].

Fragmented DNA is released into the bloodstream during the growth and expansion of tumors [25, 26]. Increased plasma cirDNA concentrations have been observed in several types of cancer, leading to the assumption that the concentrations of plasma cirDNA may serve as biomarker for early cancer detection and diagnosis, or for prognosis by monitoring or potentially predicting the response to therapies [27-31]. Increased plasma cirDNA concentration have also been reported in many other non-oncogenic pathologies as well (e.g., trauma, sepsis, myocardial infarction, etc.; for review see [32]). One major challenge in the assessment of cirDNA is the complex origin of the nucleic acids that are present in circulation. Nucleic acids are released from tumors as well as from normal cells through several cellular mechanisms such as apoptosis, necrosis, exosome-mediated release, and shedding from macrophages after the absorption of necrotic cells [33-36]. The use of animal models injected with tumor cells (“xenografts”) enables the concomitant study of cirDNA in bodily fluids and tissue samples, and has been suggested as a useful model system to examine the origin and variations of cirDNA upon experimental interventions [37-40]. Notably, increasing concentrations of cirDNA in OSA patients have been identified and were positively correlated with disease severity, suggesting that cirDNA may reflect pathogenic changes that may be relevant to disease severity or to its associated morbidities such as cancer [41].

As tumors develop, cells undergo major epigenetic changes [42]. Epigenetic aberrations can be also used to detect and characterize malignant growth [43], particularly by characterizing the scope and magnitude of epigenetic DNA alterations in plasma, serum and other bodily fluids [44]. In this study, we describe the first comprehensive cirDNA analysis in cancer associated to intermittent hypoxia in an animal model, as a hallmark of OSA in humans. Using xenografted mice exposed to IH and control conditions, we here show a significant increase on plasma cirDNA amounts, which is associated with the severity of the tumor. Moreover, we provide a large-scale epigenetic profiling of plasma cirDNA in xenografted animals exposed to intermittent hypoxia. We used system biology approaches to genomic variations associated with IH exposure. Lastly, we applied single-locus qPCR strategies to study candidate loci in plasma cirDNA and genomic DNA from tissue and blood samples.

## RESULTS

### Tumor growth and invasion in xenografted mice exposed to intermittent hypoxia

**Figure 1:**
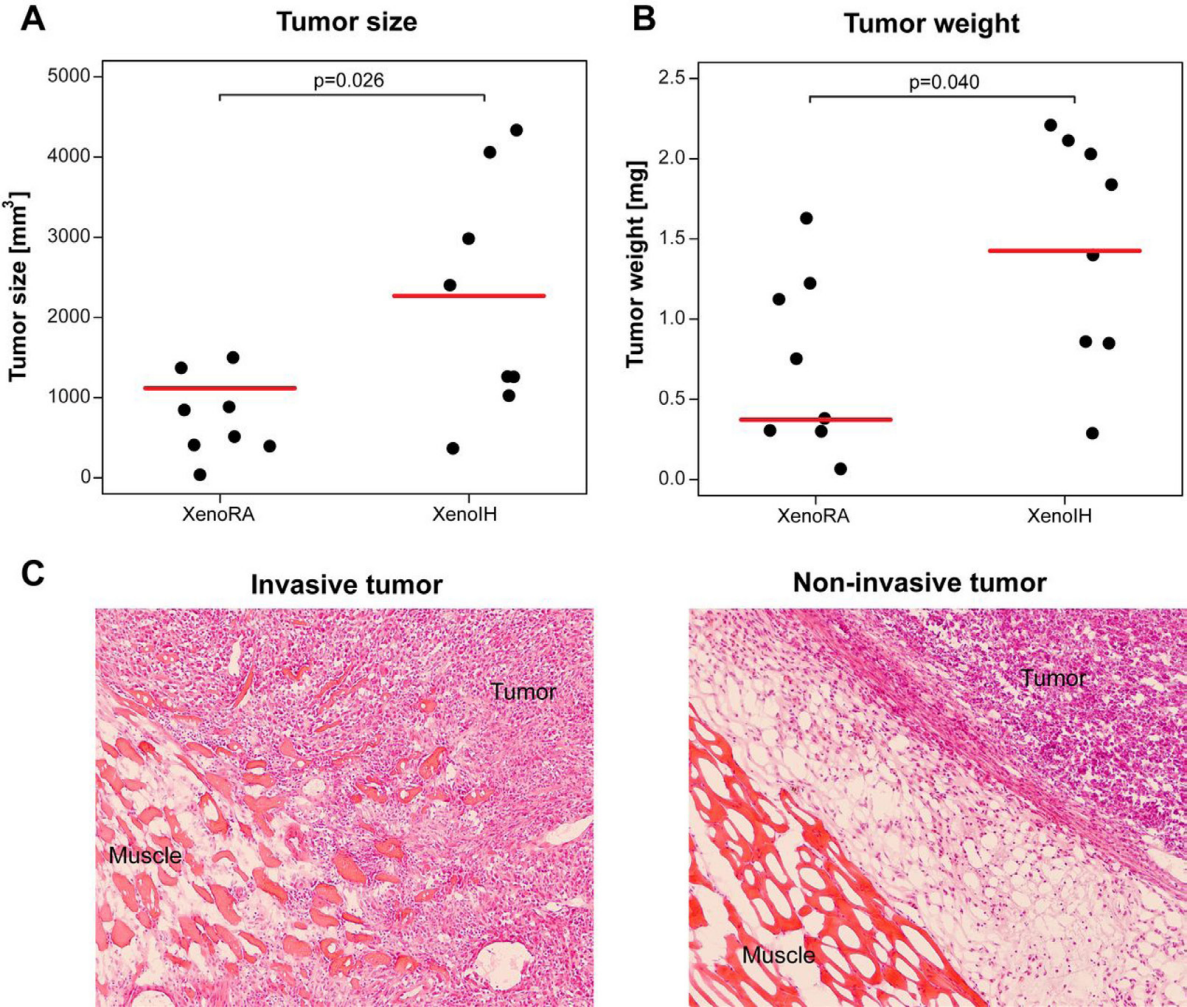
Effects of IH exposure on xenografted tumors Tumors in animals exposed to IH grew significantly larger than those in control mices. Y-axis depicts the volume (panel A) and weight (panel B) of the tumors assessed at the time of sacrifice (4 weeks after injection). Horizontal red lines correspond to the median size for each group. Panel C illustrates tumor invasiveness as observed upon IH exposures (left panel) and non-invasive tumor, as observed mainly in RA conditions (right panel).

Tumor size and weight in the XenoIH group were higher compared to the XenoRA group (Figures 1A and 1B, respectively). We found significant differences for tumor size (median sizes were 745.78 mm^3^ and 2211.43 mm^3^ for XenoRA and XenoIH, respectively; t = −2.66, df = 8.61, p-value = 0.026; Welch Two Sample t-test), and tumor weight (median weights were 0.72 mg and 1.45 mg for XenoRA and XenoIH, respectively; t = −2.27, df = 13.16, p-value = 0.040). Invasion towards the skeletal muscle was observed in all tumors in the XenoIH group (n=8), but only in 3 out of 8 tumors in the XenoRA group (p=0.025, Fisher's Exact test) (Figure 1C).

### Quantification of circulating DNA (cirDNA) in plasma

**Figure 2:**
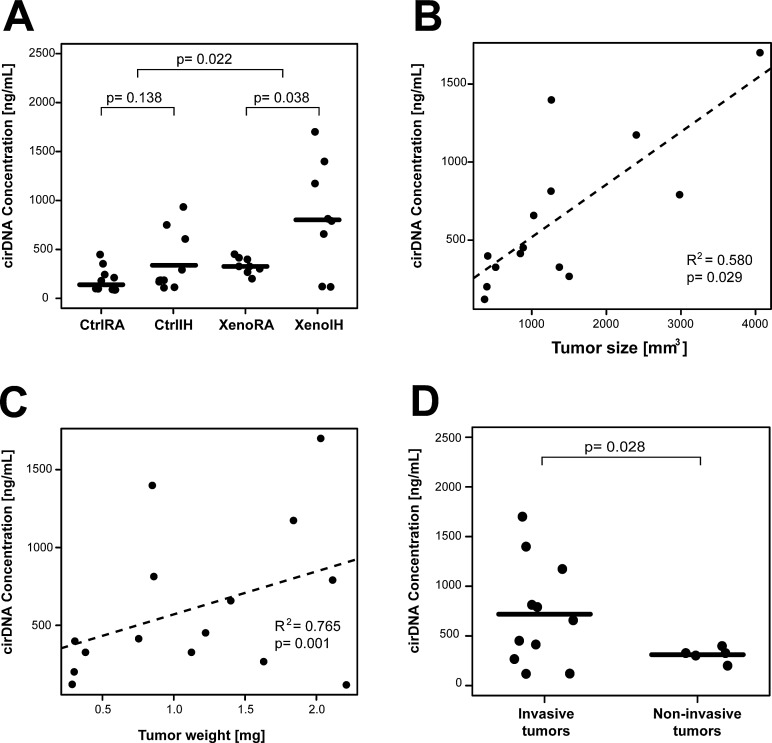
Plasma cirDNA concentration in xenografted and control mice under IH and RA conditions A) Mouse bearing the tumors showed significantly increased plasma cirDNA amounts. IH exposures are associated with elevated plasma cirDNA concentrations in xenografted (XenoIH and XenoRA groups) and control (CtrlIH and CtrlRA groups). Horizontal lines correspond to the mean size for each group. B) and C) Plasma cirDNA showed a significant positive correlation with tumor size (Panel B) and weight (Panel C). Dashed lines depict the trend line for each correlation. D) Plasma cirDNA is significantly elevated in invasive tumors compared to non-invasive tumors. Plasma cirDNA concentration were assessed by qPCR. p-values < 0.05 are considered significant (t-test).

Mean cirDNA amounts were highest in IH-exposed mice, particularly in XenoIH mice, with a significant group effect (F (3, 32) = 6.89, p=0.001; one-way ANOVA) (Figure 2A). Tukey post-hoc comparisons indicated significant differences between the XenoRA and XenoIH groups (M=-510.62, 95% CI (−940.83, −80.42), p=0.015). Pairwise comparison showed that mice bearing the tumors (XenoRA and XenoIH groups together, mean cirDNA concentration = 591.28 ng/mL plasma) had significantly higher plasma cirDNA concentration than those not carrying the tumors (CtrlRA and CtrlIH together, mean cirDNA concentration = 271.44 ng/mL plasma) (t = −2.47, df = 21.05, p-value = 0.022, Welch Two sample t-test). Exposure to IH resulted in increased plasma cirDNA concentrations in xenografted mice (mean cirDNA concentrations: XenoIH=846.59 ng/mL plasma, XenoRA=335.96 ng/mL plasma; t = −2.53, df = 7.30, p-value = 0.038), and in mice not carrying the tumors, although the latter differences were not statistically significant (mean cirDNA concentrations: CtrlIH=352.76 ng/mL plasma, CtrlRA=190.12 ng/mL plasma; t = −1.59, df = 12.14, p-value = 0.138).

Significant correlations emerged between the concentration of plasma cirDNA and tumor weight (R^2^=0.580, p=0.029; Pearson's product-moment correlation test) (Figure 2B) and tumor size (R^2^=0.765, p=0.001) (Figure 2C), but not with the weight of the mice (R^2^=-0.134, p=0.437) (Figure S1). Furthermore, we found that mice bearing invasive tumors (mean concentration 718.65 ng/mL plasma) had significantly higher plasma cirDNA concentrations than those bearing non-invasive tumors (mean concentration 311.06 ng/mL plasma) (t = 2.53, df = 10.79, p-value = 0.028; Welch Two Sample t-test) (Figure 2D).

### Epigenomic profiling of plasma cirDNA

We selected 3 samples that best represented each group according to the biological variables (Table S1) and interrogated these samples using microarrays. Data quality control showed that all microarray results were robust, with equivalent densities of signal intensity. All microarrays were included in posterior analyses (n=3 per group) (Figure S2).

**Figure 3:**
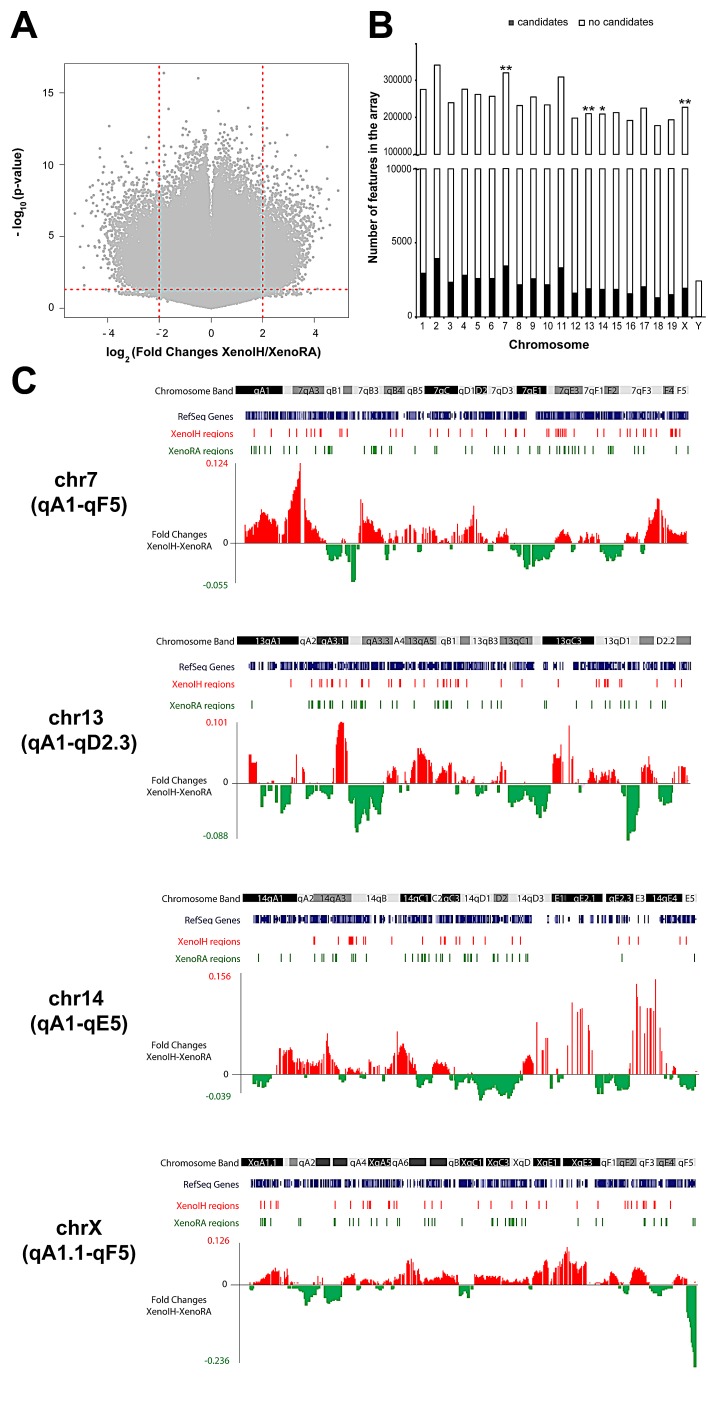
Large-scale cirDNA modification profiling using promoter microarrays A) Volcano plot of microarray data. The x-axis represents fold changes differences between the groups, with coefficients expressed in the log_2_ scale. Samples with increased microarray signals in XenoIH and XenoRA groups had positive and negative coefficients, respectively. The y-axis represents the −log_10_-transformed p-values. The horizontal dashed red line depicts the cutoff value for the p-value (−log_10_(p< 0.05)=1.3). The vertical dashed red lines depict the cutoff vallues for the fold changes (log_2_(4)=2). B) Chromosomal distribution of microarray features showing differential cirDNA modification between the groups. Stacked bars show the number of features that showed (black) or not showed (white) differential cirDNA modification per chromosome. Significance level have been determined by Fisher test (**: p<0.01; *: p<0.05) C) Genome browser images showing the distribution of differential cirDNA modification in chromosomes 7, 13, 14 and X. Chromosome bands are shown in black, grey and white scale. RefSeq genes are shown in blue. Regions of differential cirDNA modification in XenoIH and XenoRA groups are shown as red and green bars, respectively. Red and green peaks correspond to fold changes higher in the XenoIH and XenoRA groups, respectively.

Of the 4,106,240 mouse-genome features on the microarray, 46,589 features showed statistically significant differences (p-value<0.05; One-way ANOVA) and fold changes higher than 2 (Figure 3A). Analysis of chromosomal distribution showed that features showing differential microarray signal were overrepresented on chromosomes 7 (p=0.009, OR=0.96, 95% CI = 0.92-0.98, Fisher's exact test), 13 (p=0.005, OR=1.07, 95% CI = 1.02-1.12), 14 (p=0.04, OR=1.05, 95% CI = 1.00-1.10) and X (p=0.002, OR=0.93, 95% CI = 0.88-0.97) (Figure 2B, Table S2). Figure 2C shows the distribution of the candidate regions across those chromosomes. We identified two chromosomal bands containing significantly overrepresented clusters of features with decreased microarray signal in the Xeno IH group compared to the XenoRA group: 7qB3 (p=0.002, OR=0.00, 95% CI = 0.00-0.50) and XqF5 (p=0.039, OR=0.00, 95% CI=0.00-1.13)(Table S2).

**Figure 4:**
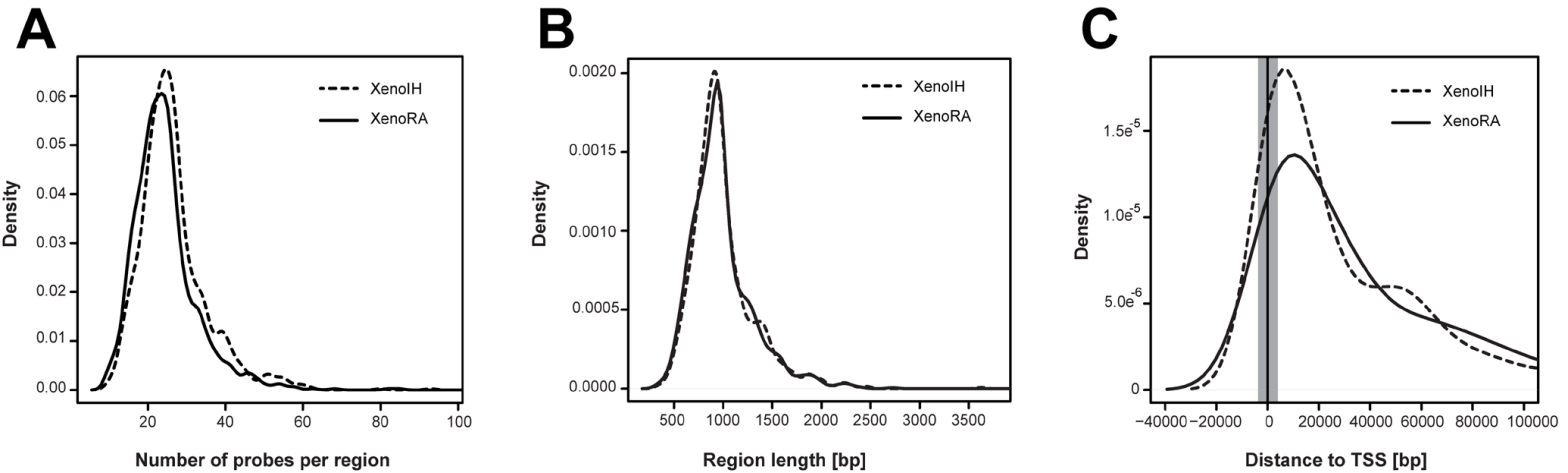
Characterization of regions showing differential cirDNA modifications Adjacent microarray features showing equivalent differences between the XenoIH and XenoRA groups were combined, according to the MAT algorithm. A) Density plot of the number of features per region in each group. B) Density plot of the length of the candidate region in each group. C) Distribution of the distance to TSS in each group. The distance from the begining of each region to the closest TSS are shown in the X-axis. Gray-shaded area depicts the segment designed as “TSS-associated”(± 2 kbp from TSS). Dashed and solid lines represent the XenoIH and XenoRA groups, respectively.

To detect genomic regions showing differential cirDNA modification, adjacent probes showing equivalent differential cirDNA modification were grouped. We identified 2,094 differentially modified regions, with 1,053 and 1,041 regions showing higher cDNA modification in XenoIH and XenoRA groups, respectively (Table S3). We did not detect significant differences in the length (996.25 ± 320.84 and 1002.27 ± 578.88 nucleotides, respectively) or number of features (26.51 ± 8.82 and 26.61 ± 12.66 features, respectively) between regions displaying higher cirDNA modifications in the XenoIH or XenoRA groups (p=0.557; Wilcoxon rank sum test) (Figure 3A and 3B). Among the differentially modified regions, 1,568 were associated to annotated RNA transcripts, with 1,406 associated to mRNA transcripts and 107 to non-coding RNAs (ncRNA). Regions were mapped associated to the Transcription Start Site (TSS) (n=231; association was defined as TSS ± 2 kb) or to the gene coding portion (n=1,337) of the gene (Table S3). We found significant differences in the distribution of the distance to the TSS between regions with higher cirDNA modifications in the XenoIH (n=723; mean distance to TSS=48.6 ± 84.4 kbp) and the XenoRA groups (n=845; mean distance to TSS=68.8 ± 115.0 kbp) (p=6.41 × 10^−6^; Wilcoxon rank sum test) (Figure 4C). The number of TSS-associated regions was significantly overrepresented among the regions with higher cirDNA modification in the XenoIH group (n=131/723) compared to those in the XenoRA group (n=100/845) (p=5.8 × 10^−4^ OR=1.65, 95% CI = 1.23-2.21, Fisher's exact test). To identify the putative regulatory elements located within those regions, we selected the top 10 motifs in each group and compared them against databases of DNA binding factors. We identified 53 DNA binding factors, of which 13 were unique for the regions with high cirDNA modification in the XenoIH group, 28 for those in the XenoRA group and 15 were shared between the candidate lists. Table 1 shows the top 10 transcription binding site regions found in each group.

**Table 1:** DNA binding factors associated to regions showing differential cirDNA modification

Both groups		XenoIH group		XenoRA group	
Factor name	p-value^a^	Factor name	p-value^a^	Factor name	p-value^a^
ZNF263	2.05×10^−9^	Nkx3-1_primary	8.99×10^−5^	Nr5a2	1.87×10^−9^
Zfp281_primary	4.45×10^−6^	Bapx1_2343.1	8.99×10^−5^	RREB1	1.91×10^−7^
Zfp740_primary	9.65×10^−6^	Nr2f2_primary	3.08×10^−4^	Esrrb	1.40×10^−4^
Spi1	1.15×10^−5^	EWSR1-FLI1	3.87×10^−4^	ESR1	3.32×10^−4^
EGR2	6.49×10^−5^	JUN	5.05×10^−4^	Stat6	3.60×10^−4^
SP2	6.51×10^−5^	Tbp_primary	6.14×10^−4^	Bcl6	3.76×10^−4^
Ets1	1.24×10^−4^	FOXP1	8.39×10^−4^	Foxj1_primary	5.13×10^−4^
Mtf1_secondary	2.05×10^−4^	Tcf12	8.51×10^−4^	E2F1	5.18×10^−4^
E2F3	2.13×10^−4^	Srf_secondary	8.99×10^−4^	Zfp105_primary	5.67×10^−4^
SP1	3.09×10^−4^	Osr2_secondary	9.75×10^−4^	E2F4	6.35×10^−4^

a p-value from hypergeometric test

We further assessed the Gene Ontology (GO) groups overrepresented in regions displaying differential cirDNA modifications (Table S4). Among the regions showing higher cirDNA modification in the XenoIH group, we observed enrichment in categories associated to glutamate metabolism (i.e. GO:0014065~phosphoinositide 3-kinase cascade; 45.9 FC) and transport mechanisms (i.e. GO:0051938~L-glutamate import; 23.0 FC; GO:0032983~kainate selective glutamate receptor complex; 23.2 FC). Conversely, in regions with cirDNA modification that were lower in XenoIH, we observed high enrichment in categories associated to establishment of location (i.e. GO:0010159~specification of organ position; 24.8 FC), cyclic nucleotide metabolism (i.e. GO:0004115~3′,5′-cyclic-AMP phosphodiesterase activity; 18.7 FC) and cellular organization (i.e. GO:0016010~dystrophin-associated glycoprotein complex; 9.5 FC; GO:0005605~basal lamina with 8.8 FC).

### Single locus DNA modification analysis of candidate regions

**Figure 5:**
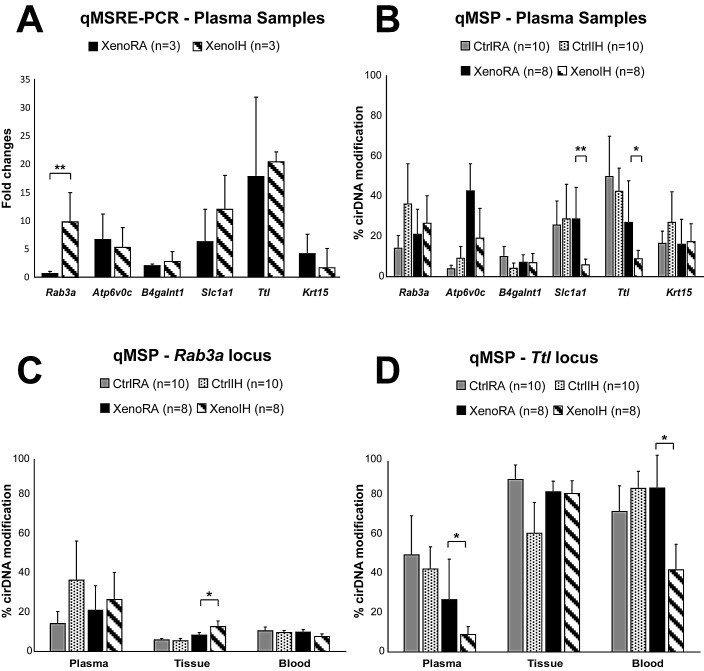
Single locus cirDNA modification analysis A) Plasma cirDNA modifications were assessed in six loci by qMSRE-PCR in the same samples used in the microarray. Y-axis shows the XenoIH/XenoRA fold changes. B) cirDNA modification by qMSP results in all plasma samples. Y-axis represents the % of cirDNA modification. C) and D) qMSP results in plasma, tissue and blood samples for the *Rab3a* and *Ttl* loci, respectively. Solid black, dashed bars, solid gray and dotted bars represent the XenoRA, XenoIH, CtrlRA and CtrlIH groups, respectively. The height of the bars corresponds to the mean values. Error bars are SE. Significance level was determined by F-test (**: p<0.01; *: p<0.05).

We selected 6 loci (Table 2, Figure 5A) displaying significant differential cirDNA modification in the microarray analysis (*Rab3a*, *Atp6v0c*, *B4galnt1*, *Slc1a1*, *Ttl* and *Krt15*) and whose corresponding genes have been reported as associated to lung cancer phenotypes (Table 2). The qMSRE-PCR assays contained, at least, one site for the restriction enzyme used in the microarray analysis. We observed high intragroup variation in the cirDNA modification enrichment across all studied loci. Noteworthy, while the MATscores show the cumulative DNA modification effects at the restriction sites of the three enzymes across extended DNA fragments captured by adjacent probes in the tiling microarray, qMSRE-PCR assays cover much shorter DNA fragments (around 100 bp) enabling the assessment of DNA modification only at one restriction site. Therefore, the precise CG positions driving the cirDNA modifications observed by microarray might have not been targeted in the verification effort. Despite the biological and methodological caveats, we detected one locus (*Rab3a*) showing significant cirDNA modification differences between the XenoRA and XenoIH groups (mean enrichment: XenoRA=0.7 ± 0.3 FC, XenoIH=9.83 ± 5.2 FC; p=0.008, F-test) (Table 2; Figure 5A).

**Table 2:** Single locus cirDNA modification analysis

*qMSRE-PCR in plasma samples from microarray analysis*							
Loci	Enrichment XenoRA^a^	Enrichment XenoIH^a^	p-value Xeno^c^
*Rab3a*	0.66 ± 0.33	9.82 ± 5.13	**0.008**
*Atp6v0c*	6.62 ± 4.52	5.31 ± 3.43	0.729
*B4galnt1*	2.04 ± 0.29	2.81 ± 1.73	0.055
*Slc1a1*	6.31 ± 5.7	12.01 ± 6.03	0.944
*Ttl*	17.83 ± 14.07	20.41 ± 1.73	0.766
*Krt15*	4.19 ± 3.38	1.69 ± 3.38	0.286

*qMSP in all plasma samples*							
Loci	% mod. CtrlRA^b^	% mod. CtrlIH^b^	% mod. XenoRA^b^	% mod. XenoIH^b^	p-value Ctrl^d^	p-value Xeno^c^	p-value Graft^e^
*Rab3a*	14.18 ± 6.19	36.22 ± 19.94	21.03 ± 12.54	26.53 ± 13.73	**0.030**	0.973	0.450
*Atp6v0c*	3.82 ± 1.81	9.18 ± 5.85	42.63 ± 13.63	19.15 ± 14.75	0.062	0.903	**0.001**
*B4galnt1*	9.93 ± 5.15	4.21 ± 2.50	7.28 ± 3.60	6.90 ± 4.71	0.102	0.395	0.880
*Slc1a1*	25.72 ± 11.97	28.66 ± 17.37	28.68 ± 15.87	5.92 ± 2.74	0.102	**0.005**	0.316
*Ttl*	49.80 ± 20.24	42.59 ± 11.36	26.91 ± 20.78	8.97 ± 4.14	0.289	**0.025**	0.668
*Krt15*	16.56 ± 6.14	27.02 ± 15.19	16.18 ± 12.36	17.41 ± 8.95	**0.048**	0.361	0.863

*qMSP in tissue samples*							
Loci	% mod. CtrlRA^b^	% mod. CtrlIH^b^	% mod. XenoRA^b^	% mod. XenoIH^b^	p-value Ctrl^d^	p-value Xeno^c^	p-value Graft^e^
*Rab3a*	5.92 ± 0.69	5.55 ± 1.05	8.36 ± 1.22	12.60 ± 2.83	0.417	**0.042**	**4.6×10^−4^**
*Ttl*	90.81 ± 7.48	60.98 ± 15.76	84.44 ± 5.57	83.65 ± 6.56	0.305	0.796	0.060

*qMSP in blood samples*							
Loci	% mod. CtrlRA^b^	% mod. CtrlIH^b^	% mod. XenoRA^b^	% mod. XenoIH^b^	p-value Ctrl^d^	p-value Xeno^c^	p-value Graft^e^
*Rab3a*	10.51 ± 1.94	9.74 ± 1.03	9.95 ± 1.22	7.61 ± 1.30	0.323	0.916	0.814
*Ttl*	72.13 ± 15.37	86.19 ± 8.84	86.47 ± 16.84	42.14 ± 13.32	0.923	0.709	0.301

Statistically significant differences (p<0.05, F-test) are in bold.

a Mean enrichment of the digested fraction compared to the undigested sample (mean ± SE).

b Mean % DNA modification (mean ± SE).

c p-value of XenoIH vs. XenoRA comparison

d p-value of CtrlIH vs. CtrlRA comparison

e p-value of Xeno vs. Ctrl comparison, without differentiating by IH-exposure

Next, we extended the analysis to all mice included in the study. We quantified the cirDNA modification in the 6 loci in plasma cirDNA (Table 2 and Figure 5B) as well as genomic DNA samples from tumor tissues and peripheral blood cells (PBC) (Table 2 and Figures 5C and D). Quantitative methylation specific PCR (qMSP) assays contained at least one restriction site for the enzymes used in the microarray and qMSRE-PCR assays. Similarly to the observations by qMSRE-PCR, intragroup variation in plasma cirDNA samples was high. We detected two loci (*Slc1a1* and *Ttl*) showing significant cirDNA modification differences between the groups (*Slc1a1* locus: mean cirDNA modification: XenoRA= 28.7 ± 15.9 %, XenoIH= 5.9 ± 2.8 %; p=0.005; *Ttl* locus: mean cirDNA modification: XenoRA= 26.9 ± 20.8 %, XenoIH= 9.0 ± 4.1 %; p=0.025) (Figure 5B). We quantified the DNA modification values in two loci (*Rab3a* and *Ttl*; Table 2 and Figure 5C and D, respectively) in genomic DNA from tissue and PBC samples. The observed intragroup variation was lower than in plasma cirDNA. For the *Rab3a* locus, we detected significant DNA modification differences in tissue genomic DNA concordant with those observed in plasma cirDNA (mean cirDNA modification: XenoRA= 8.4 ± 1.2 %, XenoIH= 12.6 ± 2.8 %; p=0.042), but no differences were detected in PBC genomic DNA (mean cirDNA modification: XenoRA= 9.9 ± 1.2 %, XenoIH= 7.6 ± 1.3 %; p=0.916) (Figure 5C). Conversely, DNA modification percentages in the *Ttl* locus were equivalent for the XenoRA and XenoIH groups in tissue genomic DNA (mean cirDNA modification: XenoRA= 84.4 ± 5.6 %, XenoIH= 83.6 ± 6.5 %; p=0.796), but DNA modification in PBC genomic DNA was higher in XenoRA than in XenoIH (mean cirDNA modifications: XenoRA= 86.5 ± 16.8 %, XenoIH= 42.1 ± 13.3 %; p=0.709) in concordance with plasma cirDNA results, though the evident differences did not reach statistical significance (Figure 5D).

## DISCUSSION

In this study, we combined the benefits of a murine xenograft model with sensitive detection using real-time PCR methods and epigenetic profiling using high-density microarrays to study cirDNA in tumors exposed to IH, a hallmark of OSA.

Although elevated amounts of plasma cirDNA have been widely reported in the majority of cancer types, their application as biomarkers has been questioned, primarily because of the high inter-patient variation within cases and controls [45, 46]. We found that the amount of cirDNA in plasma was significantly increased in xenografted mice when compared to those not bearing the tumors (Figure 2A). We observed some intra-group variation, even when our experimental setup enabled the control of phenotypic variables that can covariate with shedding of DNA to circulation (i.e. age, sex, genetic background, etc.) or technical variables for the cirDNA handling (i.e. time to cirDNA isolation and cirDNA isolation batches), which could not be readily controlled in many studies using clinical samples. When analyzing possible covariates, we only found significant correlation of plasma cirDNA concentration with tumor size, weight and invasiveness, but not with the weight of the animal bearing the tumor or technical parameters. Our findings suggest that inter-individual variation in cirDNA shedding might be rather related to biological features of the tumor upon IH exposures. In particular, we found that exposure to IH during sleep was associated to increased plasma cirDNA in both xenografted and control mice (Figure 2A). These findings concur with reports elevated plasma cirDNA amount in OSA patients [41]. Furthermore, we have recently reported that two of the major components of OSA – sleep fragmentation and IH – promote more aggressive tumor biological characteristics [23, 24]. While the results of the present study consolidate these previous findings, further studies with clinical samples are warranted to investigate a putative biomarker application for cirDNA quantitation among cancer patients with and without concurrent sleep disorders, particularly considering the strong emerging epidemiological evidence linking adverse cancer outcomes in the presence of OSA [1, 2, 47].

Epigenetic DNA modifications (mainly, cytosine methylation and hydroxylmethylation), histone modifications and non-coding RNAs have been demonstrated as fundamental molecular mechanisms for the establishment of oncogenic phenotypes and tumor progression [42]. Furthermore, sensitive detection of epigenetic marks in cirDNA have been shown as potential biomarkers [44] and some of them are already being applied in clinical diagnostic assays (e.g., *SEPT9* DNA methylation for early detection and screening of colorectal cancer [48, 49]). Large-scale cirDNA modification analysis using high density microarrays or deep-sequencing enables the evaluation of thousands of loci in parallel to generate molecular signatures for diagnostics [50], but also enables the evaluation of variation at the epigenomic level. Here, we identified more than 2,000 regions showing differential cirDNA modifications between xenografted tumors exposed to IH or RA conditions (Figure 3A and Table S3). These regions were associated with more than 1,400 annotated mRNA transcripts and over 100 ncRNAs, suggesting a major role of epigenetic processes in the modulation of the tumor phenotype by IH-exposure.

We consequently applied system biology approaches to identify possible associations of these regions to major molecular mechanisms of genome regulation. Regions with higher cirDNA modification upon IH exposure were preferentially associated with regulatory elements proximal to TSS, while regions with decreased cirDNA modification were preferentially associated to distal regulatory elements (Figure 4C and Table 1). Several molecular models have been proposed in which the interplay between DNA modification and the binding of transcription factors (TFs) at promoter and enhancer regions regulate the expression of the cognate genes (reviewed in [51]). Our findings support further investigation on the precise epigenetic regulation of TSS-associated and distal regulatory elements in sleep disorders. Gene ontology analysis revealed an enrichment of genes involved in glutamate metabolism and transport among genes associated to regions gaining cirDNA modification upon IH-exposure during sleep. Concordant with our findings, it has been shown that cells grown under hypoxia depend on the reductive carboxylation of glutamine-derived α-ketoglutarate for *de novo* lipogenesis and tumor growth [52]. Moreover, it has been demonstrated that the glutamate-transporter SLC1A5 is essential for cell growth in lung cancer [53] and that the intake of L-glutamine regulates mTOR signalling [54].

We detected clusters of variation of microarray signals in the XenoIH group in chromosomes 7, 13, 14 and X (Figures 2B and 2C). In the microarray-based method, decrease of signals in large regions may be due to a loss of cirDNA modification, but also to genome loss by deletions of chromosome re-arrangements [50]. Partial losses in chromosomes 7 and 14 have been reported in mouse lung adenocarcinoma cell lines [55]. By analyzing signal clustering in chromosomal bands, we identified regions of significant loss of signal at 7qB3 and XqF5 loci. Although there is no information available on chromosomal rearrangements in murine lung tumor cell lines, synteny analysis revealed orthologous regions in human (15q15.3 and Xp22.2 for 7qB3 and XqF5, respectively), which are associated to malignant phenotypes in the lung. For example, loss of heterozygosity (LOH) at 15q15 has been reported in lung carcinomas [56]. In turn, the Xq22 region harbors several putative tumors suppressor genes which are inactivated by mutations in lung cancer cell lines and primary tumors (i.e. *GRPR* [57], *VEGFD* [58],and *MID1* [59]).

Analysis at single loci using quantitative approaches revealed high heterogeneity in cirDNA modification levels among plasma samples belonging to the same group (Figure 5). This is a common trend in epigenetic studies using cirDNA, which may hinder the transfer of basic research findings to clinical applications [60-62]. There are two main factors that may explain the observed heterogeneity. First, plasma cirDNA has a complex origin [35, 63, 64], since not only tumor cells, but also epithelial cells and circulating cells in blood can shed their DNA into circulation. Second, different cellular types and cells at different degree of differentiation have different epigenetic profiles, even when they belong to the same tissular structure (i.e. organ or tumor) [65]. The use of the xenografted model enabled the verification in tissue as well as blood samples from the same animal to further investigate the origin of the variation. Two loci showing significant differences in plasma cirDNA modifications illustrate the complexity of the plasma cirDNA profiles. Differences in the *Rarb3*a locus were also detected in tissue samples, but the difference was not present in PBC samples (Figure 5C), suggesting that the cirDNA profile of this locus represents that found in the tumor and DNA modification profiles in blood cells will not mask the results. On the contrary, differences in the *Ttl* locus did not represent those in the tissue, but rather reflected those found in the blood samples (Figure 5D).

In summary, we have shown that exposures to IH during sleep mimicking those routinely encountered in moderately severe OSA patients are accompanied by increases in the shedding of cirDNA into circulation, in both tumor and non-tumor-injected mice, with the presence of tumor further increasing cirDNA. Futhermore, our large-scale profiling approach revealed that cirDNA carries epigenetic modifications that may characterize specific cell populations. Furthermore, the intrinsic high variability of cirDNA methylation within the tumor suggests that some tumor cell populations may preferentially release their DNA upon IH exposures.

## Methods

### Animals, hypoxic exposures, and epithelial lung tumor model

C57BL/6J male mice (7-week old) were acquired from Jackson Laboratories (Bar Harbor, ME). IH exposures and tumor characterization were performed as previously described [23]. In brief, mice were subjected to IH with alternating cycles of 90 seconds (6% FIO_2_ followed by 21% FIO_2_, 20 cycles/hour) for 12 hours/day during daylight followed by 21% FIO2 for the remaining 12 hours. The control group was exposed to continuous circulating 21% FIO_2_ (RA). Mice were pre-exposed during 2 weeks to either RA or IH, and half of the mice were then randomly selected and injected with 1 × 10^5^ TC1 murine lung tumor cells in the left flank. Every 3 days, tumor volume was estimated. After 4 weeks from tumor injection, mice were sacrificed and tumors excised and weighed. All experimental procedures were approved by The Institutional Animal Care and Use Committee of the University of Chicago.

### Plasma cirDNA and genomic DNA isolation

Blood and tissue samples were collected after sacrifice and immediately processed. The plasma fraction was separated by centrifugation and cirDNA was isolated using the QIAmp Nucleic Acid isolation kit (Qiagen, Valencia, CA). Blood and tissue samples were lysed using Proteinase K digestion and genomic DNA isolated using standard phenol/chloroform extraction.

### cirDNA quantification

cirDNA amount in each plasma sample was quantified using SYBR-green based quantitative PCR (qPCR) [38]. A 150 bp assay was designed located in the intron 2 of the *Kras* gene. The qPCR reaction consisted of 10 uL plasma cirDNA, 1× ABI master mix containing Taq polymerase, dNTPs, SYBR green dye and ROX as passive dye (Life Technologies, Carlsbad, CA, USA) and 200 nM of specific primers (Table S5). Amplification and analyses were performed using the 7500 System (Applied Biosystems, Foster City, CA). cirDNA quantity was calculated by CT extrapolation to a calibration curve built with murine DNA.

### cirDNA modification profiling

Several biochemical modifications have been reported at cytosine residues, namely methylation (5-mC), hydroxymethylation (5-hmC), formylation (5-fmC) and carboxylation (5-cmC) [66], with 5-mC being the most widely studied, and commonly referred as “DNA methylation”. Although multiple methods of epigenetic analysis have been developed in the last decades (reviewed in [67]), not all approaches are suitable for cirDNA epigenetic analysis due to the small amount of starting material. Whereas antibody-based detection methods (i.e., MeDIP and hMeDIP) are specific for 5-mC or 5-hmC, they require relatively large starting DNA amounts, and are therefore unfeasible for plasma cirDNA analysis. Therefore, we used a bisulfite-based and methylation-sensitive enzymatic restriction approach that recognizes modified Cs, but does not differentiate between 5-mC and 5-hmC [68]. To maintain a precise terminology, we will therefore apply the term ‘DNA modification’ in the description of the experiments.

Large-scale cirDNA epigenetic modification profiles were assessed in plasma cirDNA samples from xenografted mice exposed to IH (XenoIH group, n=3) or to RA (XenoRA, n=3) conditions, according to previously described methods [50]. Briefly, universal DNA adaptors were ligated to the ends of cirDNA fragments, followed by digestion with DNA modification-sensitive enzymes (*HpaII*, *HinP1* and *HpyCH4IV)*. cirDNA fragments that survived enzymatic hydrolysis were amplified by adaptor-mediated PCR. The enriched differentially cirDNA modified fraction was fragmented, biotin-labeled, and hybridized on Affymetrix GeneChip Mouse Promoter Array 1.0R (Affymetrix, Santa Clara, CA) and scanned, according to manufacturer's protocol. The array consisted of over 4.6 million probes tiled to interrogate over 28,000 mouse promoter regions.

### Microarray data analysis

GCOS 1.3 software (Affymetrix) was used to produce .cel files. Microarray raw data were deposited in NCBI's Gene Expression Omnibus (GEO) database (accession number: GSE61070). Data quality control was performed using the STARR package [69] in the R statistical environment (version 3.0.2) [70]. Data were analyzed using the Partek Genomic Suite Software (PGS) (St. Louis, MO). Signals were adjusted according to probe sequence and background corrected using the Robust Microarray method (RMA) [71]. One-way ANOVA was used to detect probes showing differential cirDNA modification between the groups. The significance level was set on p<0.05 and fold changes higher than 2 (Figure 3). Model-based analysis of tiling-arrays (MAT) [72] was used to identify regions of differential cirDNA modification by combining adjacent probes showing significant differences between the groups. A sliding window of 500 bp was set, according to the average size of the fragments produced in the amplicon preparation step [50]. Data were visualized by importing it to the UCSC Genome Browser (http://genome.ucsc.edu, NCBI36/mm8 assembly, which corresponded to the building of the microarray). Transcript association and chromosomal distribution were determined using the LiftOver tool to the latest available mouse genome annotation (GRCm38/mm10 assembly; last accessed August 2014). Genomic motifs were identified using the Multiple Em for Motif Elicitation tool (MEME) [73] and compared them against databases of DNA binding factors using the TOMTOM motif comparison tool [74]. Gene ontology analysis was performed using the Database for Annotation, Visualization and Integrated Discovery (DAVID) version 6.7 [75].

### Single locus cirDNA epigenetic analysis by DNA modification-sensitive restriction quantitative PCR (qMSRE-PCR)

Fifteen nanograms of purified amplicon were subjected to real-time PCR, using specific primers (Table S5) and the same SYBR-green conditions used for cirDNA quantification. Adaptor-mediated PCR products from the undigested samples were used as reference to calculate the enrichment (ΔCt_target_). A fragment within the *Gapdh* locus, which did not contain any CG position, was used as calibrator (ΔCt_calibrator_). Fold change (FC) enrichment was calculated using the equation: FC=(2^(ΔCt_target_-ΔCt_calibrator_)).

### Single locus cirDNA epigenetic analysis by quantitative Methylation Specific PCR analysis (qMSP)

cirDNA and genomic DNA samples were bisulfite-treated using the Epitect kit (Qiagen) and amplified using the whole bisulfitome amplification kit (Qiagen). Bisulfite treated and amplified cirDNA (10 ng) was subjected to locus-specific amplification using qMSP primers [76] (Table S5) and the same SYBR-green conditions used for cirDNA quantification. Fragment surrounding each qMSP assay in which primers did not contain any CG site were used as reference (ΔCt_sample_)*. Sssi* methylated DNA was used as a calibrator (ΔCt_methylated)_. Percentage of methylation was calculated using the equation: %Me=(2^(ΔCt_sample_-ΔCt_methylated_))*100).

## SUPPLEMENTARY MATERIAL, FIGURES


