# Supplementary material for: Tumor circulating DNA profiling in xenografted mice exposed to intermittent hypoxia
Source: Oncotarget. 2014 Nov 16;6(1):556–69. doi: 10.18632/oncotarget.2785 (PMC4381615; doi:10.18632/oncotarget.2785)
Supplement: Supplementary file 1 [file oncotarget-06-556-s001.pdf]

Tumor circulating DNA profiling in xenografted mice exposed to intermittent hypoxia

Supplementary Material

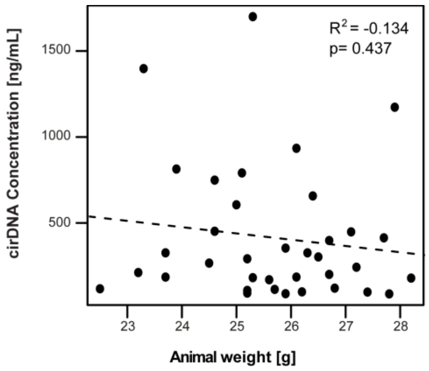

Figure S1: Plasma cirDNA modification concentration is not correlated with the mice weight. Dashed line depicts the trend line for the correlation. Plasma cirDNA concentrations were assessed by qPCR.

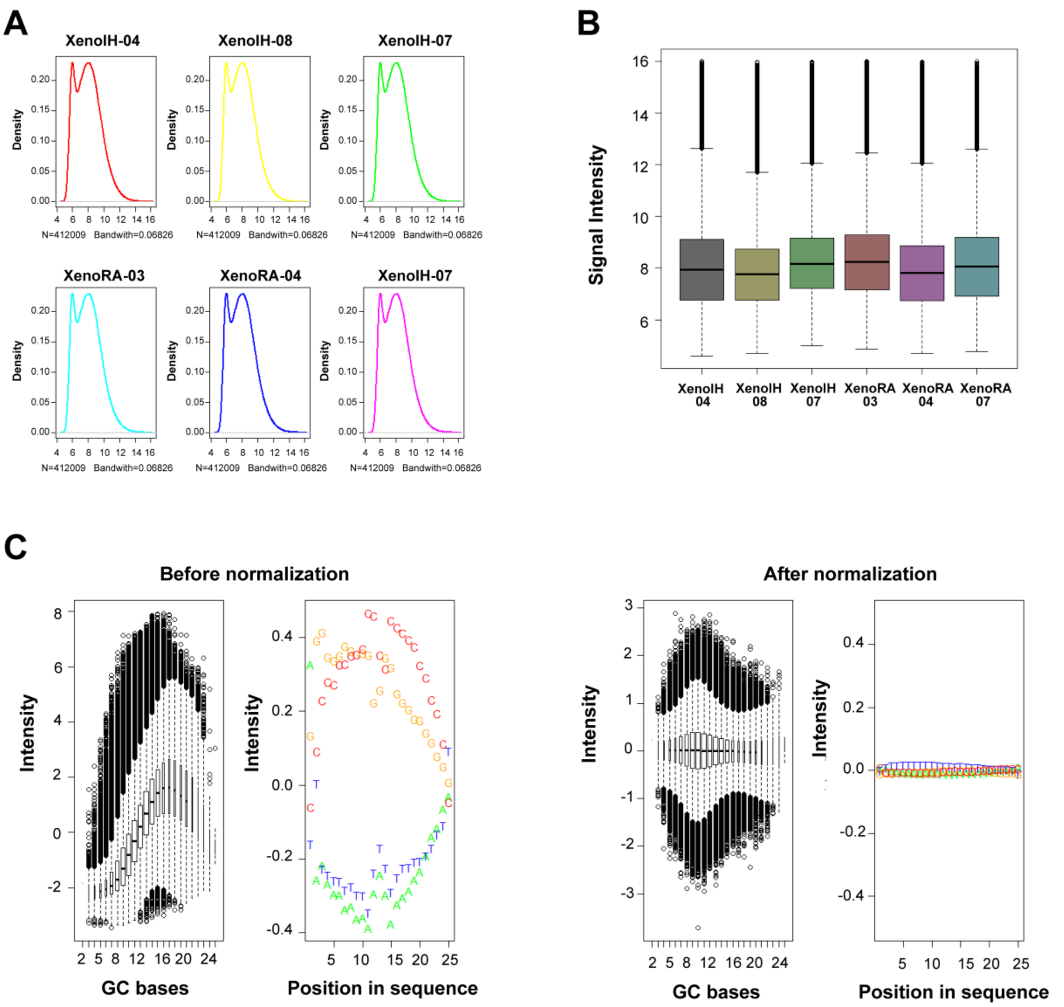

Figure S2: Quality control of microarray data. All arrays showed equivalent results and could be used for downstream analyses. A) Density plots of microarray signal distribution for each sample. Upper panels correspond to XenoIH group (XenoIH-04, XenoIH-07 and XenoIH-08). Lower panels correspond to XenoRA group (XenoRA-03, XenoRA-04and XenoRA-07). B) Box-plots showing the distribution of signal intensity for each sample before normalization. C) Correction of CG-content bias after normalization. Left and right panels show the distribution of signal intensities according to CG content before and after normalization, respectively.

Legends for supplementary tables

Table S1: Phenotypic characteristic of xenografted and control mice exposed to IH and RA conditions.

Table S2: Chromosomal distribution of cirDNA modified features.

Table S3: Annotation of regions showing differential cirDNA modifications in XenoIH and XenoRA groups.

Table S4: Gene ontology enrichment in genes associated with regions showing differential cirDNA modification.

Table S5: Primer sequences for cirDNA quantification and single locus cirDNA modification analysis.
